# Machine learning-driven Diabetes Health Tracer (DHT): Optimizing prognosis using RaSK_GraDe and RaSK_GraDeL models

**DOI:** 10.1371/journal.pone.0327661

**Published:** 2025-10-21

**Authors:** Muhammad Noman, Maria Hanif, Abdul Hameed, Muhammad Babar, Basit Qureshi

**Affiliations:** 1 Department of Software Engineering and Artificial Intelligence, Iqra University, H-9, Islamabad, Pakistan; 2 Department of Computer Science, University of Management and Technology, Lahore, Pakistan; 3 Robotics and Internet of Things Laboratory, Prince Sultan University, Riyadh, Saudi Arabia; 4 College of Computer and Information Sciences, Prince Sultan University, Riyadh, Saudi Arabia; King Saud University, SAUDI ARABIA

## Abstract

Diabetes mellitus presents a significant global health challenge, particularly in regions like Pakistan, India, and Bangladesh. Machine learning (ML) techniques offer promising solutions for diabetes prediction, surpassing traditional methods in reliability and efficiency. This research conducts a comparative analysis of ML algorithms including Random Forest (RF), Decision Tree (DT), Support Vector Machine (SVM), K-nearest neighbors (KNN), Gradient Boosting (GB), RaSK_GraDe (Proposed Voting), and RaSK_GraDeL (Proposed Stacking). Evaluation is performed using datasets, such as PIMA Indian, Frankfurt Hospitals Diabetes, RTML with Insulin, and the proposed Diabetes Health Tracer (DHT) dataset comprising 2877 observations with nine features. Data pre-processing techniques address missing values, outliers, normalization, and class balancing (SMOTE), enhancing model robustness. Hyperparameter tuning via cross-validation and Random Search optimizes model performance. Additionally, ensemble methods—Voting Classifier (RaSK GraDe) and Stacking Model (RaSK GraDeL with Logistic Regression) are applied, achieving notable accuracies of 98.03% and 98.55%, respectively, on the DHT dataset. The study underscores ML’s potential in diabetes prediction, advocating for personalized treatment and healthcare management advancements.

## 1 Introduction

Diabetes mellitus (DM) is a chronic medical condition caused by high blood glucose level due to the body’s inability to produce enough insulin or effectively use the insulin it produces. Diabetes currently stands as one of the most deadly global diseases that people are afraid of nowadays. It has two main types: type 1, which commonly occurs in children mediated by immune mechanism, and type 2, which occurs later in life due to malfunctioning or diseases of the pancreas [1]. The other two types include gestational diabetes occurs during pregnancy and its symptoms disappears after the pregnancy process, and prediabetes the blood glucose level always stand above normal range.

This health epidemic extends its influence worldwide, presenting a significant challenge for nations, particularly those in the process of development such as Pakistan, India and Bangladesh [2]. According to certain research findings, compared to non-immigrant populations, which had a prevalence of 11.6%, South Asian countries had a high rate of diabetes, with patients from the country of Sri Lanka having the highest prevalence (26.8%), followed by those from the country of Bangladesh (22.3%), Pakistan’s (19.6%), India as well (18.3%), and Nepal have (16.5%) [2]. Diabetes affects an estimated 537 million adults worldwide between the age of 20 to 79. This diabetes rate will globally increase up to 643 million in 2030, increasing to 783 million by 2045 [3]. Diabetes disease instantaneously caused 6.7 million death in 2021 according to World Health Organization (WHO). Moreover, the amount of time spent on diabetes in health organization has increased by around 316% in the last 15 years [4].

Diabetes short-term symptoms are caused by high glucose level include polyuria, polydipsia, weight loss, blurred vision and sometimes polyphagia. Long-term complications of diabetes include heart-attack, partial paresis, foot ulcers, loss of vision, sexual dysfunction, and cerebrovascular disease [5]. Diabetes is one of the leading causes of chronic kidney disease (CKD). The people with diabetes develop CKD with ratio of 40%, and the number of new cases of CKD in people with type 2 diabetes increases up to 74% from 1990 to 2017 [5]. According to a Global healthcare expenditure due to diabetes the cost will grow from 966 billion U.S dollar to just over one trillion U.S dollar between 2021 and 2025 [6].

A well-known way of treating diabetes was first used three decades ago is self-monitoring of blood glucose (SMBG) using finger-stick blood samples [7,8]. With the aforementioned method, diabetics use finger-stick glucose metres to prick their finger skin three or four times a day to monitor their blood glucose levels in an intrusive manner. The idea is to measure blood glucose concentrations at various intervals and modify insulin dosage, food, and exercise to keep blood glucose levels within normal ranges. Nevertheless, if the estimation of insulin intake is based on a small number of SMBG samples, this method may be deceptive in addition to being difficult and uncomfortable. As a result, there is a chance that the plasma glucose levels will rise over normal. Continuous glucose monitoring (CGM), which offers the most information about variations in blood glucose concentration throughout the day and helps diabetes patients make the best treatment decisions, was launched as a solution to the aforementioned issue. This method uses tiny wearable sensors or systems to continually monitor blood glucose levels by tracking the amounts of glucose in the blood all day long. These systems may be non-invasive, minimally invasive, or intrusive. Moreover, it is possible to classify CGM systems into two groups: real-time systems and retrospective systems [9]. The arrival and accessibility of numerous cutting-edge continuous glucose monitors (CGMs) electronic devices and systems present new chances for diabetic individuals to easily manage their blood sugar levels. The majority of contemporary CGM typically use a minimally invasive technique to continuously measure the interstitial fluid (ISF) to calculate and record the patient’s current glycemic status every minute. These systems/devices just breach the skin’s outer layer without actually piercing any blood vessels, and are considered minimally invasive. Moreover, there exist non-invasive techniques, such as using electromagnetic radiation to measure blood glucose levels by passing it through the skin and into the body’s blood vessels [10]. diagnosis of diabetes may not always be possible using the traditional techniques, due to factors such as, poverty, and distant location of hospitals from people living in villages. Therefore, the popularity of artificial intelligence techniques play an important role in healthcare centers along with improvement in technologies. Especially, Machine learning (ML) algorithms due to reliability and robustness. ML algorithms give a persuasive result in very less time as compared to old classical methods.

Therefore, the objective of this research is to create a new dataset by merging different datasets, proposed a system that can easily predict diabetes in real-time, and to do a comparative analysis of the performance of different ML algorithms such as Random Forest (RF), Decision Tree (DT), Support Vector Machine (SVM), KNN, and Gradient Boosting (GB). The algorithms are implemented on each dataset to do binary classification of diabetes. Their performances are assessed by various evaluation metrics namely accuracy, recall, F1-score, ROC-curve, AUC-score, and Precision.

The remainder of this paper is structured as follows: Sect 2 presents the background and a review of related work. Sect 3 outlines the proposed methodology in detail. Sect 4 provides experimental results and a comparative analysis with state-of-the-art approaches. A concise discussion of the system’s performance is provided in Sect 5, and concluding remarks are given in Sect 6. Table 1 lists the abbreviations used throughout the paper for clarity and reference.

**Table 1:** List of acronym and abbreviations.

Acronym	Definition
ADASYN	Adaptive synthetic sampling
ANN	Artificial Neural Network
AUC	Area under the ROC Curve
CKD	chronic kidney disease
CGM	Continuous glucose monitors
D1	Datasets 1 (PIMA)
D2	Dataset 2 ( FHD )
D3	Dataset 3 ( RTML-I )
DT	Decision Tree
DHT	Diabetes Health Tracer
FHD	Frankfurt Hospitals diabetes
FP	False positive
FN	False negative
GB	Gradient Boosting
IDMPF	Intelligent diabetes mellitus prediction framework
IG	Information Gain
IQR	Interquartile range
KNN	K-nearest neighbors
LOF	Local Outlier Factor
LR	Logistic Regression
MLP	Multi-layer perceptron
ML	Machine learning
MAE	Mean Absolute Error
MSE	Mean Squared Error
NB	Naive Bayes
PID	Pima Indian Dataset
ROC	Receiver operating characteristic curve
RMSE	Root Mean Squared Error
RF	Random Forest
RTML-I	RTML_With_Insulin Dataset
SMBG	Self-monitoring of blood glucose
SMOTE	Synthetic Minority Over-sampling Technique
SVM	Support Vector Machine
TP	True positive
TN	True negative

## 2 Literature review

Different types of machine learning (ML) strategies have been implemented on diabetes prediction and classification to achieve high accuracy. Some of them are explain below. In [11], The author compares the accuracy and performance of five supervised machine learning algorithms to predict diabetes. The predictive power of the DT, LR, KNN, RF and SVM methods are evaluated. Furthermore, two different datasets (PIMA and Frankfurt) are used to investigate the effect of dataset size on model accuracy, with the RF approach yielding a 97% accuracy. The impact of underfitting and overfitting on predicted results is also investigated in this work.

In [12], author presents a comprehensive guide on diabetes prediction utilizing ml models like LR, SVM, NB, RF and ensemble techniques such as XGBoost, LightGBM, CatBoost, Adaboost, and Bagging. Among ensemble metods, CatBoost emerges as the most effective, boasting an impressive accuracy rate of 95.4% compared to XGBoost’s 94.3%. Furthermore, CatBoost’s higher AUC-ROC score of 0.99. Metrices used in this study are Accuracy, sensitivity and confusion matrix.

In [13], author focuses on feature selection using KNN, RF, J48 and NB. The study’s findings demonstrate that feature selection improves models by avoiding overfitting and eliminating unnecessary data. SMOTE class balancing technique has been used. Therefore, after being assessed using metrics like the F-measure, Precision-Recall curve, and Receiver Operating Characteristic Area Under Curve, the study’s results, when compared to earlier research, demonstrate that a better outcome was obtained. When medical professionals try to identify diabetes at an early stage, this discovery may have an effect on clinical practice.

In [14], the author proposed a study of early diabetes prediction using feature selection on diabetic dataset having 2500 items with 15 attributes. DT, RF, and NB algorithms were used in the study. The highest accuracy was achieved by Naïve Bayes (82.30%). In [15], the author aims to present an ensemble machine learning model for early diabetes prediction using the PIMA dataset. While preparation techniques handle outliers and missing data, shuffle split improves accuracy. In terms of accuracy, XGBoost (XG) outperforms Random Forest (RF), AdaBoost (AB), and XGBoost (XG) (0.961 +/- 0.014). XGBoost (XG) has greater AUC (96.1%), False Negative Rate, False Positive Rate, Precision (86.6%), Sensitivity (79.8%), Specificity (94.2%), and Accuracy (89.6%) values.

In [16], the author used both deep learning (ANN) and machine learning algorithms (RF, KNN) to classify the diabetes. PIMA Indian dataset were used for the study. Feature extraction technique was carried out. By applying feature extraction, the best accuracy was achieved by ANN (75.7%).

In [17], Yadav et al.’s study from 2023 shows a substantial development in the study of diabetes mellitus and fractional-order modelling. Through the use of the Atangana-Baleanu Caputo (ABC) operator, the authors have produced a model of diabetes dynamics that is more precise and thorough. In addition to adding to the body of knowledge already in existence, this work opens the door for more studies and therapeutic applications in the area.

In [18], Parveen et al. used real world clinical data CPCSSN that contains 172,168 unique patient’s data. This review of the literature offers a thorough summary of the main topics relevant to managing longitudinal data with irregular sampling and applying machine learning approaches to prognostic model diabetes. It draws attention to the difficulties, both conventional and contemporary methods, assessment criteria, case studies, and various possibilities for future research in this area.

In [19], Manarvi et, al. took a survey based on questionnaires to collect information about the patients of diabetes. One such questionnaire was modified and translated into Arabic for use in the current study in order to survey patients at a nearby hospital. Nineteen hundred and one patients took part in this study. The outcomes of the tests are examined based on patient demographics, diagnoses, tests, and other elements of their diabetes self-management. This review of literature offers a thorough analysis of the body of knowledge regarding diabetes treatment procedures in Arabic-speaking nations, emphasising significant discoveries and suggesting areas in need of more research.

Smith et al. (2019) used random forest and logistic regression models to predict diabetes in their study. The random forest model performed better since it could handle non-linear correlations, with an accuracy of 78% and 85%, respectively. PCA and RFE were two of the methods used for feature selection, which improved model performance by finding important predictors. Although these developments, the research highlighted problems with complex model interpretability, generalisability, and data quality [20].

In [21], the author proposed a study on diabetes prediction and classification using PIMA Indian dataset. Three machine learning algorithms (Decision Tree, SVM and Naïve Bayes) have been used. The result showed that the highest accuracy was achieved by Naïve Bayes (74.28%) along with precision (75.7%), Recall (76.1%) and F1-Measure (75.8%). In [22], The author presents the Diabetes Expert System, which improves diabetes prediction through the use of Machine Learning Analytics (DESMLA). Five class balancing techniques were used by DESMLA to address the imbalance in diabetes datasets. DESMLA improves predictive accuracy by using Random Forest (RF) and Decision Tree (DT) classifiers in conjunction with thorough data pretreatment procedures. Interestingly, DESMLA works best when using Gaussian SMOTE and K-Means SMOTE approaches.

In [23], the author carried out diabetes prediction using supervised machine learning on PIMA Indian dataset. Two ML algorithms (KNN, Naïve Bayes) have been used, in which Naïve Bayes outperformed with an accuracy of (76.07%). In [24], the author uses different algorithms to predict diabetes after a rigorous experimental analysis. Compared to other algorithms, logistic regression with all features produces a better result (ACC = 84.70%) for diabetes prediction. The model performs better than the other approaches due to feature engineering to choose the right characteristics. This indicates that an 85% accuracy is obtained with the best logistic regression using certain features. In [25], a dataset including 340 occurrences and 26 characteristics is used to compare two Machine Learning methods for diabetes classification. The Bagging and Decorate Ensemble Machine Learning methods were used with WEKA software. While Decorate’s accuracy was 98.53%, Bagging’s was 95.59%. With a Kappa Statistic of 0.9214, MAE of 0.0482, and RMSE of 0.1546, bagging successfully identified 95.5882% of the occurrences. Furthermore, for Bagging, the TP rate was 0.956, the FP rate was 0.032, and the Specificity was 94.9%.

In [26], the author proposed a study of diabetes data classification using deep learning approach on 130-US hospital dataset. Five machine learning algorithms have been used including NB, RF, DT, SVM, and ensemble learning. The best accuracy of ML algorithm was (86%), while deep learning was (85.61%). In [27], the author uses the Pima dataset in order to accurately predict diabetes using machine learning techniques. Preprocessing methods such as feature selection, imputation of null values, scaling, and uniformity are used in combination with a number of classification algorithms, including Decision Tree (J48), NB, SVM, LR, Multilayer Perceptron, KNN, Logistic Model Tree, RF, and others. At 80.869% accuracy, the RF model gives the highest result. In [28], the author examines the use of predictive analytics in healthcare, highlighting how it can be helpful for practitioners, data-driven patient care decisions. Using a dataset of patient medical information, six machine learning methods are examined. We apply SVM, KNN, RF, DT, LR, and NB on the PIMA dataset and compare and assess each model’s accuracy and performance. By determining the best ml model for the prediction, the study seeks to help medical practitioners anticipate diabetes early on. KNN performed well among all other algorithms.

In [29], the author proposed a study of gestational diabetes on PIMA Indian dataset using Parameter-Tuned KNN. The author used grid search hyper parameter optimization technique. The accuracy was improved by 5.29% achieving the best (82.5%). In [30] , the author examines the possibility of diabetes disease through an analysis of five supervised ml models: SVM, NB, DT, RF, and KNN. After post-classification and cross-validation, the author observes steady accuracy by taking into account all risk factors in the dataset. The KNN achieves the best accuracy of 76%, while other classifiers also retain accuracy above 70%. Examining training and testing accuracy visualizations for indications of model overfitting and underfitting, the author looks at why some ML classifiers are unstable and inaccurate. The main goal of the research is to determine the best outcomes for diabetes illness prediction in terms of computing time and accuracy.

In [31], the author proposed a study of diabetes prediction using data mining technique on PIMA Indian dataset. Four methods have been used such as RF, SVM, LR, and NB. The performances were measured by confusion matrix, sensitivity and accuracy metrics. In Logistic Regression the accuracy was high as (82.46%).

In [32], the author developed the intelligent diabetes mellitus prediction framework (IDMPF), a framework for diabetes prediction. The Pima dataset was utilised by them. The achieved accuracy was 83%. However, the model gives result but not so good, so their is a way to make it better. In [33], The study’s methodology comprises parameter evaluation, prediction, ML algorithm selection, pre-processing, cross-validation, and dataset selection. Additionally, the study used 10-fold cv to divide the data into training and testing using the WEKA software. To ensure that every instance in the dataset had the same weight, a class balancer technique was used. For the PID dataset, SVM had the highest accuracy of 74.3%, while KNN and RF had the highest accuracy of 98.7% for the Germany diabetes dataset.

In [34], the author used machine learning in order to diagnose and predict diabetes, which facilitates decision-making on the management of the condition. The multilayer perceptron algorithm is the most predictively accurate of these, with an excellent area under the curve of 86%, a low mean square error of 0.19, and low rates of false positives and false negatives. The methodology includes analyzing diabetes datasets using both neural network-based and conventional classification algorithms. Performance metrics are evaluated to ascertain the efficacy of the algorithms in terms of prediction accuracy, false positive and false negative rates, and overall area under the curve. The overall related work has been shown in Table 2.

**Table 2:** Related work.

Ref	Year	ML models	Dataset	Best model	Performance Metrics
[11]	2023	DT, LR, KNN, RF, SVM	PIMA, Frankfurt	RF	Accuracy: 97%
[12]	2024	LR, SVM, RF, NB, XGBoost, LightGBM, CatBoost, Adaboost, and Bagging	early stage diabetes prediction	CatBoost	Accuracy: 954%
[13]	2024	J48, RF, NB, KNN	early stage diabetes prediction	RF	Accuracy: 98.80%
[14]	2019	DT, RF, NB	2500 items with 15 attributes	NB	Accuracy: 82.30%
[15]	2022	RF, XGBoost, AdaBoost	PIMA	XGBoost	Accuracy: 75.7% AUC: 96.1% Sensitivity: 79.8%
[16]	2019	ANN, RF, KNN	PIMA Indian	ANN	Accuracy: 75.7%
[17]	2021	DT, SVM, NB	PIMA Indian	NB	Accuracy: 78.5% Recall: 76.1% F1-Measure: 75.8%
[18]	2022	RF, DT	Diabetes datasets	DESMLA	Predictive accuracy
[19]	2023	KNN, NB	PIMA Indian	NB	Accuracy: 76.07%
[20]	2021	Logistic regression	PIMA Indian	Logistic Regression	Accuracy: 84.70%
[21]	2019	Bagging, Decorate, WEKA	Bangladesh’s dataset with 340 instances and 26 features	Bagging	Accuracy: 95.59% MAE: 0.0482 RMSE: 0.1546
[22]	2023	NB, RF, DT, SVM, Ensemble	130-US hospital	ML	Accuracy: 86%
[23]	2022	DT (J48), NB, SVM, LR, MLP, KNN, LMT, RF	PIMA Indian	RF	Accuracy: 80.869%
[24]	2018	SVM, KNN, RF, DT, LR, NB	PIMA	KNN	Accuracy: 76%
[25]	2023	Parameter-Tuned KNN	PIMA Indian	KNN	Accuracy: 82.5%
[26]	2021	SVM, NB, DT, RF, KNN	Diabetes datasets	KNN	Accuracy: 76%
[27]	2023	RF, SVM, LR, NB	PIMA Indian	LR	Accuracy: 82.46% Confusion matrix
[28]	2022	-	Pima Indian	IDMPF	Accuracy: 83%
[29]	2023	SVM, KNN, LR, RF	PIMA, Germany Diabetes Dataset	SVM and RF	RF Accuracy: 98.7% SVM Accuracy: 74.3%
[30]	2021	MLP	PIMA	MLP	Accuracy: 86% MSE: 0.19 AUC: 86%

## 3 Proposed work

In this section, we created a unique dataset by combining three distinct datasets that share similar characteristics but vary in the number of observations. Fig 2 visually depicts the framework of this methodology, containing several key steps: dataset merging, data preprocessing, dataset splitting, utilization of machine learning models like RF, DT, SVM, KNN and GB, ensemble techniques, and evaluation using various performance metrics i.e. accuracy, F1-score, recall, and precision.

**Fig 1:**
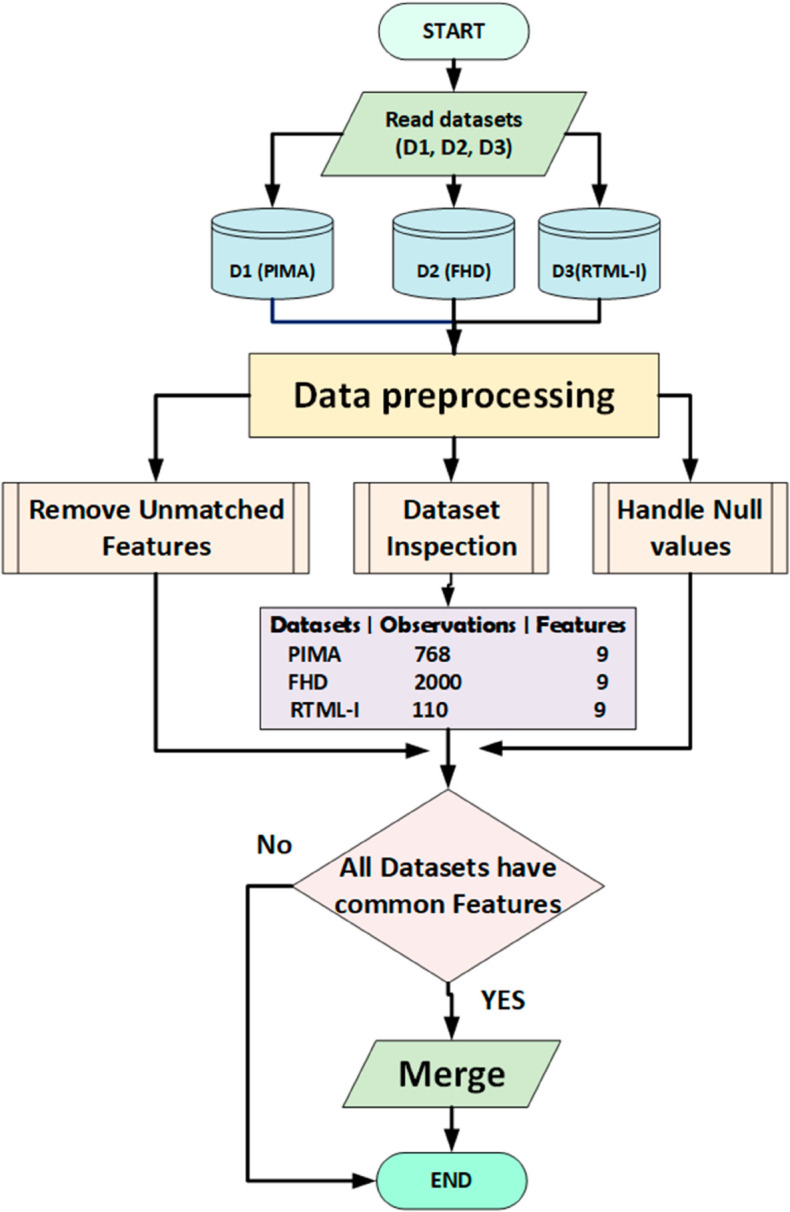
Creation of DHT (Proposed) dataset.

**Fig 2:**
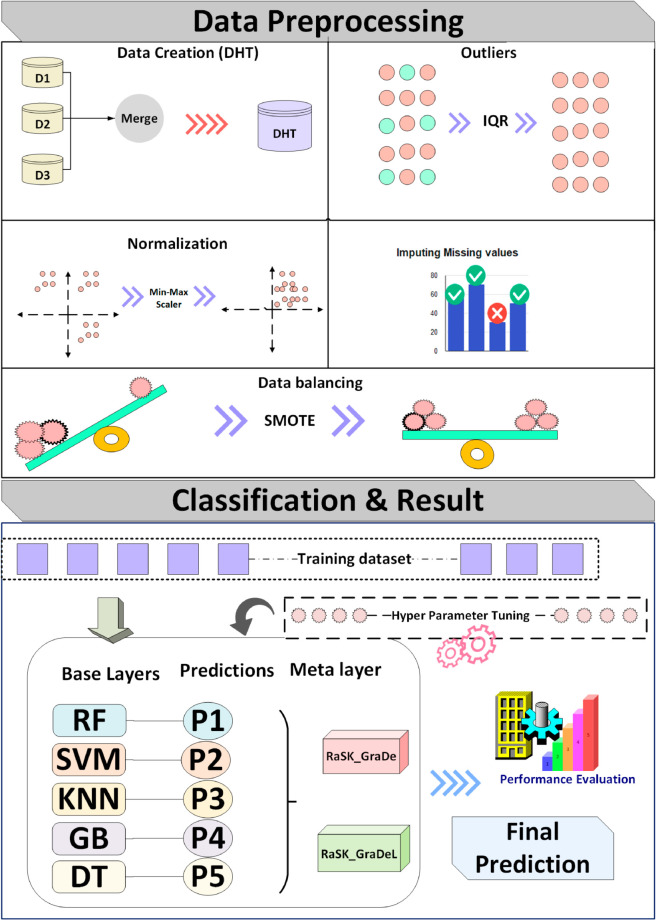
Proposed system architecture.

### 3.1 Dataset creation

In our research, we created a new dataset by merging three distinct datasets: a) PIMA Indian [35] b) Frankfurt Hospitals Diabetes [36] and c) RTML with Insulin [37], which is Obtained from 103 female individuals of Rownak Textile Mills Ltd, Dhaka, Bangladesh. These datasets are publicly accessible on platforms like Kaggle and github. Initially, we imported these datasets and examined their contents individually to understand their characteristics and patterns.

The PIMA Indian dataset comprises 768 observations with 9 features, while the Frankfurt Hospitals Diabetes dataset includes 2000 observations with the same features as the PIMA Indian dataset. The RTML with Insulin dataset contains 110 observations with 8 features. Notably, one feature was missing in the RTML with Insulin dataset, which we addressed by employing a machine learning technique called median imputation.

After removing any redundant variables, we merged these datasets to create a proposed dataset called Diabetes Health Tracer (DHT) as shown in Fig 1. Subsequently, we utilized this dataset for training models to predict diabetes as described in Algorithm 1.

**Algorithm 1. Dataset creation.**

**Require:** Read Datasets (PIMA, FHD, RTML-I)

1: **for all** *d* **in** Datasets **do**

2:   **for all** feature_name **in** *d* **do**

3:    **if** feature_name is not common **or** feature_name is

Unnamed **then**

4:     Remove feature_name from *d*

5:    **end if**

6:   **end for**

7: **end for**

8: Declare variable Combined_dataset

9: **for all** *d* **in** Datasets **do**

10:   Combined_dataset = merge(d, Combined_dataset)

11: **end for**

12: **return** combined_dataset =0

The process of creating proposed DHT dataset has been explained below:

1. Reading the datasets: We have read all three datasets (PIMA, FHD, RTML with Insulin) for the merging process.
2. Inspection: In the second step we inspect datasets one by one to check their behaviours and patterns.
3. Remove Unmatched features: In this step we remove the features which are not aliened or not common in all three datasets. There was only one features in “RTML with Insulin” dataset which has no name so we remove it.
4. Merging datasets: After removing Unnamed feature we merged all three datasets into DHT (proposed) dataset using concatenation technique, and saved the DHT (proposed) dataset for future use.

**Fig 3:**
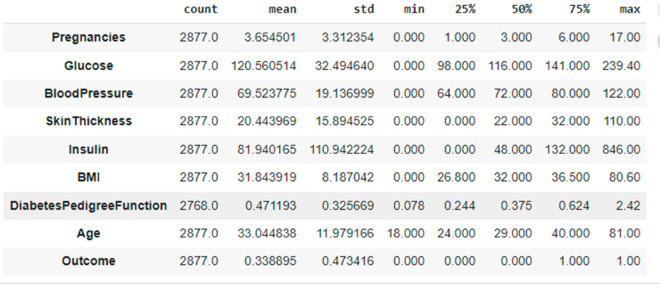
Descriptive analysis of DHT.

The proposed DHT dataset comprises 9 attributes, including one target variable. The Diabetes Health Tracer (DHT) dataset is publicly available under the Apache License 2.0. The dataset can be accessed at Diabetes-Health-Tracer-DHT-Dataset. Also the repository link is present in the reference section at [38]. It consists only numeric data, with a total of 2877 observations. The target variable indicates two classes (0,1). Table 3 gives a summary of the attributes, types, and values of the proposed DHT dataset. Descriptive analysis of DHT using Measures of central tendency, frequency and standard deviation as been shown in the Fig 3.

**Table 3:** Diabetes health tracer dataset description.

S.No	Attributes	Type	Values	Descriptive meaning of attributes
1	Pregnancies	Numeric	0-17	How many times individual gets pregnant?
2	Glucose	Numeric	0-199	Using an oral glucose tolerance test to measure glucose concentration
3	BloodPressure	Numeric	0-122	Blood pressure (mm Hg)
4	SkinThicnkess	Numeric	0-99	Thickness of skin (mm)
5	Insulin	Numeric	0-846	2-Serum insulin (mu U/ml) at 2 hours
6	BMI	Decimal	0-67.1	Body mass index of individuals (kg/(m)^2^)
7	GlucosePdegreeFunction	Decimal	0.08-2.42	Glocoe percentage
8	Age	Numeric	21-81	Age in years of individuals
9	Outcome	Numeric	1 , 0	Target variable

### 3.2 Data preprocessing

Data preprocessing is a technique that is required to prepare the raw data for another processing procedure [39]. Preparing data is a prerequisite step to develop a reliable predictive model. It is the process of making the data suitable to train a machine learning model. Following are the preprocessing techniques that we applied on the data to make it suitable for ML models and ensemble techniques:

#### 3.2.1 Imputation of missing values.

**Fig 4:**
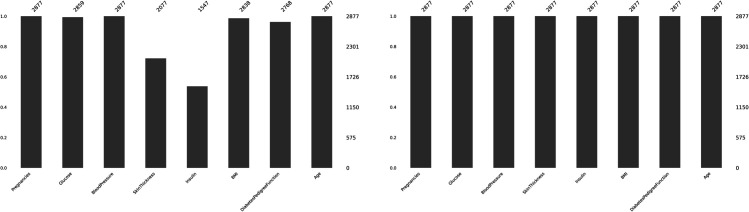
Missing value matrix: Missing values of overall dataset.

We observed that the dataset has missing values as shown in the Fig 4. Missing data causes machine learning models to malfunctioning like overfitting or under-fitting due to which accuracy of algorithms effected. Features containing missing values has been shown in Table 4. It is clearly shown in the Fig 4 that the features like Glucose, Insulin, GlucosePedgreeFunction, SkinThickness, and BMI have some missing values. We use imputation method to handle this problem. We have used Median to remove missing values from the dataset. The median is the middle value of a dataset when it is ordered from lowest to highest. If there is an even number of values, the median is the average of the two middle values. Unlike the mean, the median is not affected by outliers, making it a more reliable measure for skewed distributions. The formula for median imputation is given below.

$${\hat{x}}_{i}=\left\{ \begin{array}{cc} \text{median} & \text{if }x_{i}\text{ is missing}\\ x_{i} & \text{otherwise} \end{array}\right.$$ (1)

$${\hat{x}}_{i}=\left\{ \begin{array}{cc} \frac{\text{median}(x_{\left(n/2\right)}),\text{median}(x_{\left(n/2+1\right)})}{2} & \text{if }x_{i}\text{ is missing}\\ x_{i} & \text{otherwise} \end{array}\right.$$ (2)

where ${\hat{x}}_{i}$ is the imputed value for observation *i*, and *x*_*i*_ is the original value. The median is calculated based on the available non-missing values. Equation (1) is commonly used for the imputation of missing values having odd numbers while on the other hand, equation (2) is used for for handle the missing values having even numbers.

**Table 4:** Features with number of missing values.

Features	Number of missing values
Glucose	18
Insulin	1330
GlucosePedgreeFunction	109
SkinThickness	800
BMI	39

#### 3.2.2 Normalization.

The Min-Max scaling technique is applied to the non-binary features in the dataset in order to scale these features. The Min-Max scaler scales the data within the range (0,1). The formula is given below.

$$X_{\text{min-max}}=\frac{X-\min(x)}{\max(x)-\min(x)}$$ (3)

The Equations (3) is most commonly used for normalization of the data and is adapted from [40]. Normalised feature values can be understood as representing the original value’s range between the initial minimum and maximum, from 0 percent to 100 percent. [40].

#### 3.2.3 Outlier detection and removal.

Some of the features in the dataset have outliers. The presence of outliers makes a model biased and affects its predictive performance. In this study, the inter-quartile- range (IQR) method is applied to remove outliers from the dataset. In this method, the data points that are outside the range between the 1st quartile (Q1) and the 3rd quartile (Q3) are considered outliers and removed by replacing their values with the median value of the specific column. In the IQR method, the threshold is calculated by the following equation:

$$T_{\text{lower}}=Q1-1.5(\text{IQR})$$ (4)

$$T_{\text{upper}}=Q3+1.5(\text{IQR})$$ (5)

IQR=Q3−Q1 (6)

**Fig 5:**
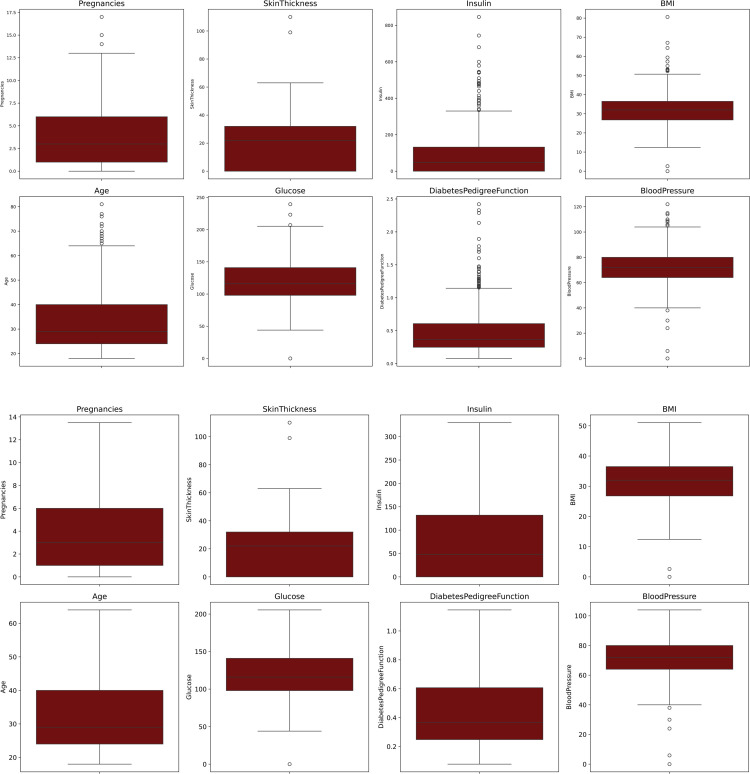
Features of DHT dataset with outliers.

Eqs (4), (5), and (6) are utilised to identify and eliminate outliers from the data; they were derived from [41]. Here Q1 and Q3 refer to the 1st and 3rd quartile respectively and c is the threshold which is usually set to 1.5 [41]. In Fig 5, (a) shows the outlier values of all features (Glucose, BloodPressure, Insulin, SkinThickness, BMI, GlucosePedgreeFunction, and Age). In (b) the outlier values has been removed using IQR method.

#### 3.2.4 Imbalanced dataset.

The term “imbalance” describes the dataset’s uneven class assignment. Bias in the classification results from an imbalance in data. In the dataset, this problem is apparent. We apply SMOTE class balancing techniques which has been explained below.

**Synthetic Minority Over-sampling Technique (SMOTE):**

In [42], author presented a unique technique called SMOTE “Synthetic Minority Over-sampling Technique” to increase the decision area of the minority class samples and address the problem of over-fitting. This method uses the feature space, not the data space, to create synthetic samples. Creating artificial data is used to oversample the minority class, as compared to replacing or using randomised sampling approaches. In order to improve the data space and address the lack of data in the sample distribution, it was the first strategy to add new data points to the learning dataset [42]. When classifying imbalanced data (such as minority classes), the oversampling technique is a standard procedure [43]. In the last ten years, a great deal of work has been put into it by machine learning researchers. Algorithm 2 presents the working of SMOTE.

**Algorithm 2. Synthetic Minority Oversampling Technique (SMOTE).**

**Require:** Training data

1: *Tr* is the input for the training set

2: Closest neighbour equals *p*

3: Closest neighbour after data cleansing equals *k*

**Ensure:** Following SMOTE = New_Tr, the training set

4: Get going

5: **for** *i* = 1 to *N* **do**

6:   Create artificial samples from the minority class and add

them to New_Tr

7: **end for**

8: **END** =0

**Fig 6:**
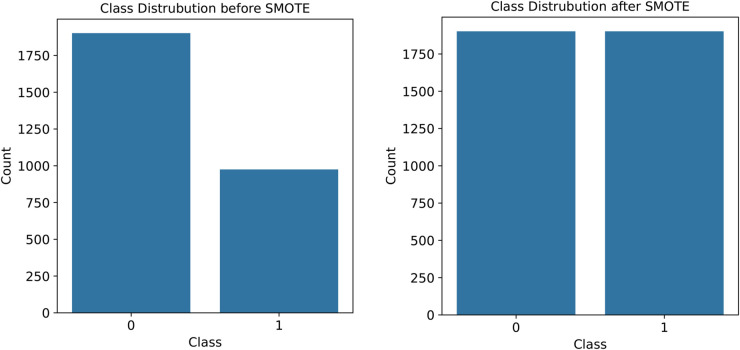
Comparison of class distribution.

The class distribution before and after the balancing techniques has been shown in Table 5. The total number of observation was increased from 2877 to 3788 due to the oversampling technique. Fig 6 visualizes the class distribution of the imbalanced dataset and balanced dataset respectively.

**Table 5:** Class distribution.

Imbalanced dataset	
**Class**	**Count values**
Total	2877
Positive 1	975
Negative 0	1902
**Balanced dataset**	
**Class**	**Count values**
Total	3788
Positive 1	1894
Negative 0	1894

**Fig 7:**
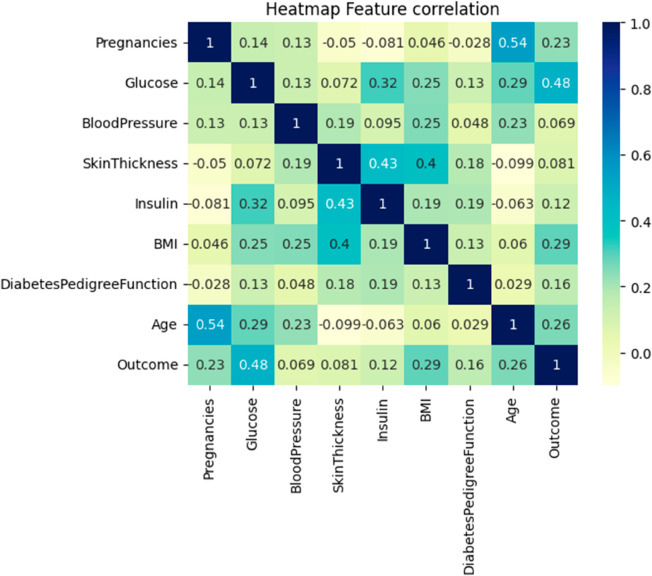
Heat map.

A popular visualisation technique for revealing patterns hidden in the data is the heatmap [44]. The Fig 7 show the correlation of the features. The heathmap shows that the target variable ‘Outcome’ is highly depend on Glucose, BMI, Age, and Insulin features.

### 3.3 Dataset splitting

In this study, the train-test splitting technique was utilised to achieve better accuracy. Train-test splitting is a classic approach in which the dataset is split into two parts. One part is used to train the models while the other one is used for testing purpose. In this study, we split the dataset into (80 20). The 80% was used for training and the 20% was used for testing.

### 3.4 Machine learning algorithms

Different types of machine learning (ML) models were used in this study. Following are some suggested supervised machine leaning (ML) models that has been used in this work.

#### 3.4.1 Decision tree classifier.

Decision trees (DTs) are a type of supervised machine learning technique that can be used for regression and classification tasks. It consists of a tree-structured classifier, in which every leaf node delivers the classification result and every node inside the tree represents the properties of a dataset [45]. A decision tree (DT) consists of two nodes: the decision node and the leaf node. Decision nodes are used to conduct actions and include some branches, whereas leaf nodes show the results of those decisions and do not contain any additional branches.It gets its name from the fact that, just like a tree, it starts at the base and spreads outward on successive branches to form a structure that resembles a tree.The leaves stand in for the options or alternatives. These decision nodes divided up the data. There are two metrics related to decision tree (DT) building: information gain (IG) and entropy/Gini-index. The two formulas for the metric computation are shown below.

$$\text{Entropy}(S)=-\sum_{i=1}^{n}p_{i}\log(p_{i})\;$$ (7)

$$\text{IG}(S,A)=\text{Entropy}(S)-\sum_{v\in \text{Values}(A)}\frac{\left|S_{v}\right|}{\left|S\right|}\cdot \text{Entropy}(S_{v})\;$$ (8)

$$\text{Gini}=1-\sum_{i=1}^{n}p_{i}^{2}\;$$ (9)

#### 3.4.2 Random forest classifier.

A type of ensemble learning, random forest (RF) is a supervised machine learning classifier. An ensemble of DTs, the majority of which were trained using the “bagging” technique, is combined to make a forest. The bagging approach’s core tenet is that mixing several learning strategies can lead to superior outcomes [46]. Using voting approaches, this supervised learning system makes a prediction about the outcome. The random forest (RF) forecasts that the final prediction will also be 1 if the majority of the forest’s trees expect that number [47]. Furthermore, a quantitative technique called random forest (RF) uses decision tree classifiers on many resamples of the dataset before averaging the results to improve prediction accuracy and avoid overfitting. When bootstrap = True, the max samples parameter sets the size of the resamples; otherwise, each tree is generated using the entire dataset [48]. The same metrics, that are employed in decision tree (DT) classifiers are also used in random forests (RF). The decision tree (DT) portion above already displays the equation for those metrics.

#### 3.4.3 Support vector machine.

Support vector machines (SVMs) are a class of supervised learning approaches that address regression issue analysis, outlier identification, and classification problems. The ability of SVMs to choose a decision boundary that minimises the distance between nearby data points across all categories sets them apart from other classification methods. The highest margin hyperbolic decision boundary or the SVM-produced decision boundary classifier is referred to as “plane and plane.” Kernel SVM and basic SVM are the two types of SVMs [49,50]. This research used the kernel SVM. To the kernel SVM, we applied the linear kernel SVM. Compared to linear kernel functions, there are fewer parameters to optimise and the majority of other kernel functions are slower. The equations that the linear kernel SVM utilises are described below [49].

$$f(X)=\sum_{i}\alpha_{i}\times K(x_{i},x)+b\;$$ (10)

In this formula, the variables *K*, *X*, and *b* represent the weight matrix to be optimised, the data to be interpreted, and the projected linear coefficient from the training or test dataset, respectively.

#### 3.4.4 K-Nearest Neighbors (KNN).

The k-nearest neighbour (KNN) algorithm is a supervised machine learning technique that is mostly used for classification. It has been widely used to forecast illnesses. The supervised KNN method makes predictions about the classification of data without labels by using the features and labels of the training data. The KNN technique can usually classify datasets using a training model that is similar to the testing query by taking into account the k nearest training data points (neighbours) and choosing the ones that are closest to the query it is testing [51]. TThe algorithm then choose which categorisation to use in the final stage using a majority voting rule. The KNN method is one of the most fundamental kinds of machine learning algorithms and is often used in classification issues due to its very flexible and easy to understand architecture. It is commonly recognised that the technique can solve regression and classification problems with data of different sizes, label counts, noise levels, distances, and contexts [51]. Distance formula (d) is used to calculate the distance measures.

$$\text{d}=\sqrt{\left({x_{2}}^{2}-{x_{1}}^{2})+({y_{2}}^{2}-{y_{1}}^{2}\right)}$$ (11)

#### 3.4.5 Gradient boosting classifier.

Gradient Boosting algorithm created for regression challenges. By repeatedly merging weak learners like decision trees into an additive approximation of a target function, it seeks to produce a strong learner. The algorithm trains models using datasets containing computed pseudo-residuals in order to minimise the predicted value of a particular loss function [52]. Regularisation is performed by shrinking, reducing tree complexity (e.g., depth), and adding hyper-parameters. Overfitting is an issue. To fine-tune the model, it is imperative to examine parameters such as learning rate, maximum tree depth, sub-sampling rate, characteristics considered for splitting, and minimum samples for node splitting. By adding regularisation hyper-parameters, Gradient Boosting mitigates the termination risk associated with perfect fitting. Because of the algorithm’s versatility, hyper-parameters like learning rate, tree depth, feature selection, and sub-sampling rate can be used for optimisation and better generalisation [52]. The mathematical equation is given below.

$$f(x)=\sum_{i=1}^{M}\beta_{i}h_{i}(x)$$ (12)

### 3.5 Ensemble learning techniques

Ensemble methods, sometimes referred to as multi-classifier systems, are one of the most significant ML research fields because they address a problem with classic ML approaches. Ensemble Classifiers are based on the basic principal that improved classification outcomes achieved by combining the predictions of several different base classifiers. Furthermore, connecting the predictions made by several base classifiers would correct errors made by each classifier and result in more accurate predictions than a single classifier [53]. We employed two distinct ensemble strategies in this work, which are covered in more detail below.

#### 3.5.1 Voting classifier.

**Fig 8:**
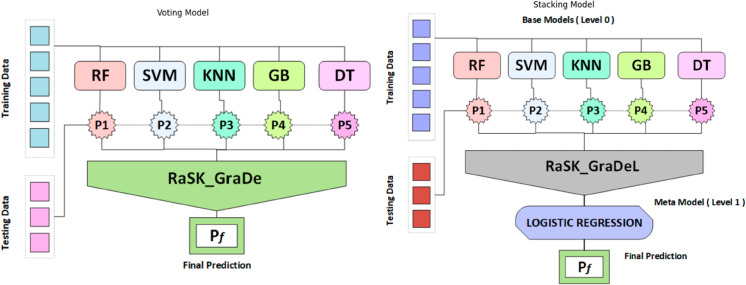
(a). Proposed voting classifier (RaSK_GraDe), (b). Proposed Stacking Model (RaSK_GraDeL).

The voting classifier is one machine learning model that trains a collection of other models. The voting classifier used the results from each classifier to forecast the output class based on the largest vote majority. Voting ensemble techniques are used by ensemble machine learning models to aggregate predictions from different models. As demonstrated in Fig 8, the voting method, which we utilised during our research, determines the class with the most votes based on the total predictions of all classifiers.

A more accurate and balanced prediction can be obtained by the voting ensemble classifier by integrating the predictions of several classifiers. Five base classifiers are used by the voting ensemble model in this study. Initially, the entire training input data set for the base model was used to train basic classifiers. Each base model’s prediction given weighted upon which the final prediction was made using soft voting mechanism. The robustness of noisy data and outliers can both be addressed using the adaptive voting ensemble classifier. The reason for this is that the same dataset is used to train different classifiers [54]. Moreover, the RaSK_GraDe (Voting Classifier) in this study of diabetes prediction system, can result in improved accuracy and robustness in prediction, making it very useful tool in the prediction of diabetes. Algorithm 3 explain the working of RaSK_GraDe (Voting Classifier).

**Algorithm 3. Proposed Voting classifier: RaSK_GraDe.**

**Require:** Dataset split into Training set $X_{\text{train}}$ and Testing set

$y_{\text{train}}$

1: **Start**

2: Establish base classifiers:

3:   Decision Tree (DT) at level 0

4:   Random Forest (RF) at level 0

5:   Support Vector Machine (SVM) at level 0

6:   K-Nearest Neighbors (KNN) at level 0

7:   Gradient Boosting (GB) at level 0

8: **for** *n* = 1 to *N* **do**

9:   Train the model *f*_*n*_ using dataset $X_{\text{train}}$

10: **end for**

11: Collect predictions from each base classifier for the test

set $X_{\text{test}}$

12: **for** each test sample *x*_*i*_ in $X_{\text{test}}$ **do**

13:   Collect predictions $p_{i1},p_{i2},\ldots ,p_{iN}$ from classifiers

$f_{1},f_{2},\ldots ,f_{N}$

14:   Aggregate predictions

15:   Store the final prediction for *x*_*i*_

16: **end for**

17: Calculate the performance of the voting classifier using the

true labels $y_{\text{test}}$

18: **END**

=0

Fig 8 presents the working of voting classifier that take predictions from base models and use them as input features for making final prediction.

#### 3.5.2 Stacking model.

Stacking approaches build an ensemble model by combining base learners, just like voting classifiers. But there are two key distinctions between voting classifiers and stacking. Firstly, unlike voting classifiers, which often use homogeneous learners (same classification algorithms), stacking typically uses heterogeneous base learners (various classification algorithms). Second, basic learners are integrated in a deterministic manner for voting classifiers. Usually, a weighted total or voting are used for this. In stacking, on the other hand, a meta learner uses a non-deterministic mechanism to combine the base learners [55].

Stacking can be done using two-layers or multiple-layers. Here in this study, we uses two-layers stacking technique. Base layer which uses five machine learning models (RF, SVM, KNN< GB, and DT) and meta-layer which uses LOGISTIC REGRESSION as a meta model. First, we trained five machine learning models as base learners on the training dataset. Secondly, we provided the prediction of each base learner models as input features to the mete-learner model. Lastly, The meta-learner model gives final prediction. Algorithm 4 explain the working of RaSK_GraDe (stacking model).

**Algorithm 4. Stacking Model: RaSK_GraDeL.**

**Require:** Dataset split into Training set $X_{\text{train}}$ and Testing set

$y_{\text{train}}$

1: **Start**

2: Establish base classifiers:

3:   Decision Tree (DT) at level 0

4:   Random Forest (RF) at level 0

5:   Support Vector Machine (SVM) at level 0

6:   K-Nearest Neighbors (KNN) at level 0

7:   Gradient Boosting (GB) at level 0

8: **for** *n* = 1 to *N* **do**

9:   Train the model *f*_*n*_ using dataset $X_{\text{train}}$

10: **end for**

11: Generate a new dataset based on the predictions

12: **for** *j* = 1 to *m* **do**

13:   Create dataset $X_{f}=\{z_{j}^{\prime },y_{j}\}$, where $z_{j}^{\prime }=\{f_{1}(z_{j}),\ldots ,f_{n}(z_{j})\}$

14: **end for**

15: Develop a meta-classifier:

16:   LR at the secondary level

17: Train the meta-classifier *F* using dataset *X*_*f*_

18: Train the complete model

19: Fit the complete model using $X_{\text{train}}$ and $y_{\text{train}}$

20: Perform the final prediction

21: **END** =0

Fig 8 visualise the working of propsed stacking model (RaSK_GraDeL) that takes input predictions from base models and then gives final prediction by using meta model (LR).

## 4 Result

### 4.1 Comparative analysis of individual datasets with proposed (DHT) dataset

We have applied machine learning algorithms on each dataset to check their performances and then compare them with our own dataset. The comparative analysis on the basis of accuracy has been shown in below Table 6. As we can seen from the Table 10, the ensemble models (RaSK_GraDe and RaSK_GraDeL) gives highest accuracy for all of the four datasets. Furthermore, The Proposed (DHT) dataset has highest accuracy score among all other datasets.

**Table 6:** Comparative analysis table.

Models	FHD	PIMA	RTML-I	Proposed (DHT)
KNN	92.79%	83.00%	94.29%	95.14%
SVM	95.64%	79.00%	94.29%	95.01%
GB	95.83%	84.50%	97.14%	96.32%
DT	91.65%	82.00%	94.29%	92.90%
RF	92.79%	86.50%	97.14%	97.11%
**RaSK_GraDe**	96.20%	89.00%	100.00%	98.03%
**RaSK_GraDeL**	96.20%	86.50%	100.00%	98.55%

**Fig 9:**
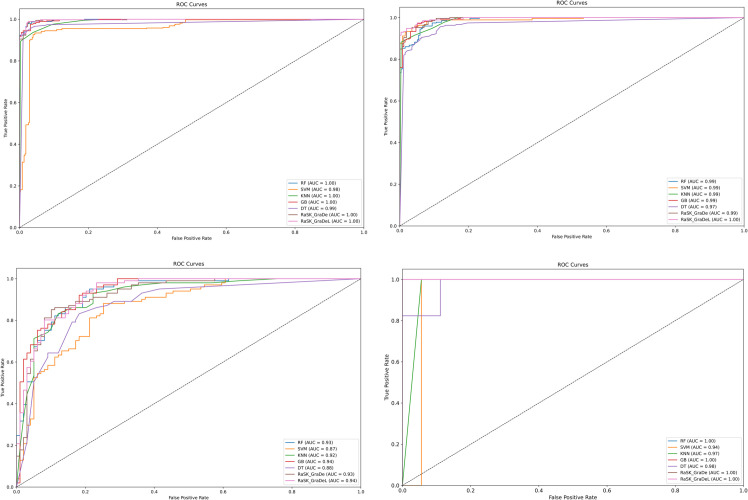
ROC_AUC curves for all four datasets.

**Fig 10:**
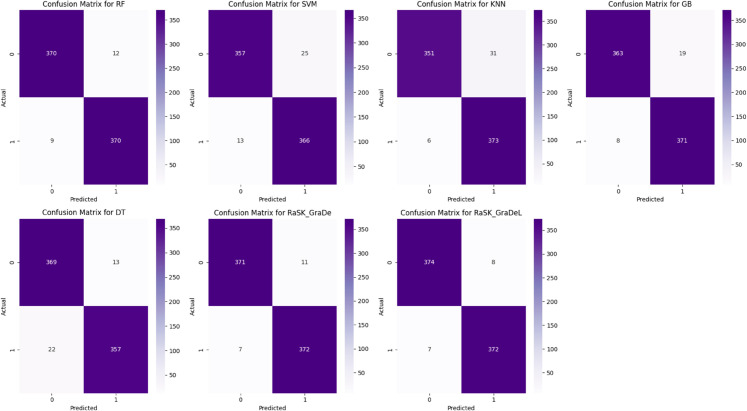
Confusion Matrices of proposed (DHT) Dataset.

Fig 9 shows the ROC_AUC curves for all four datasets along with AUC score. As it clearly shows that the ROC_AUC curve of ensemble models (RaSK_GraDe and RaSK_GraDeL) has been more accurate as compared to all other base models. While Fig 10 shows the Confusion matrix of all models for the proposed (DHT) dataset. The confusion matrix for ensemble models (RaSK_GraDe and RaSK_GraDeL) shows that it gives very high and accurate prediction.

### 4.2 Performance evaluation metrics

To determine the optimum fit, accuracy and additional statistical evaluation indicators were taken into account. ML model out of all the classifiers that were used. Based on the standards employed to assess their efficiency, every applicable supervised machine learning classifier was contrasted with one another. The most common methods for evaluating machine learning models are Precision, Recall. F1_Score and accuracy, which are produced by a confusion matrix. The ratio of successfully categorised models to all other possible outputs is known as classification accuracy. When the intended feature categories in the data are fairly similar, accuracy is a useful statistic [56].

$$\text{Accuracy}=\frac{TP+TN}{TP+FP+FN+TN}\;$$ (13)

To assess each algorithm’s performance more precisely, a number of additional evaluation measures were taken into account in addition to these four the other metrics are Recall, precision, f1-measure, and ROC Curve.Furthermore, the area under the ROC curve, or AUC, has a value between 0 and 1. Recall is a metric used to quantify how many successful outcomes machine learning (ML) systems yield [57]. The resultant score will yield the harmonic mean of accuracy and recall. To assess the F1-score, the weighted average of accuracy and recall is used [57]. Precision determines the ratio of true positives to all expected positives [57]. The terms TP, FP, and TN, FN, respectively, in the equation stand for true positive, false positive, true negative, and false negative.

$$\text{Precision}=\frac{TP}{TP+FP}\;$$ (14)

$$\text{Recall}=\frac{TP}{TP+FN}\;$$ (15)

$$\text{F1 Score}=\frac{2\times \text{Precision}\times \text{Recall}}{\text{Precision}+\text{Recall}}$$ (16)

$$\text{ROC}=\frac{TPR}{FPR}TPR=\frac{TP}{TP+FN},FPR=\frac{FP}{FP+TN}$$ (17)

**Fig 11:**
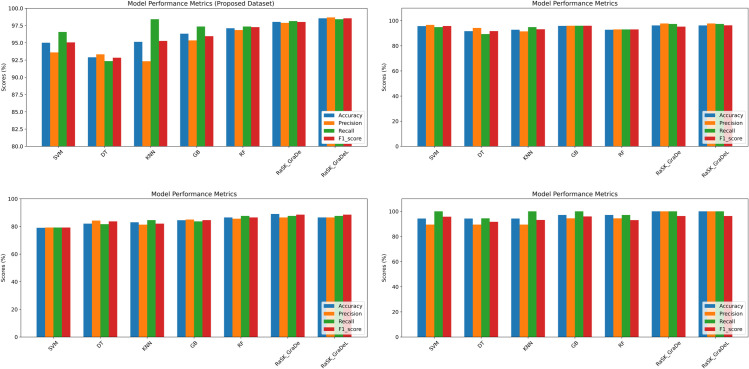
Comparative analysis of the proposed (DHT) dataset with other datasets in terms of performance metrics.

Fig 11 shows the comparative analysis of the proposed (DHT) dataset with other three datasets in terms of Accuracy, Precision, Recall, and F1_Score. As we can seen from the Fig 11 that both ensemble techniques gives highest result among all other machine learning models. Also Fig 11 depicts that proposed (DHT) dataset have high result as compared to PIMA, FHD, and RTML_I datasets.

The performances of all machine learning models and ensemble models using class balance and hyperparameter tuning are shown in Tables 7, 8, 9, and 10. The Diabetes Health Tracer (DHT) dataset, however, continuously produced the top results for all performance criteria, demonstrating the usefulness of ensemble approaches in diabetes prediction as well as the promise of the combined dataset. These tables clearly states that our ensemble models RaSK_GraDe and RaSK_GraDeL performed well on huge data as compared to small data, furthermore, these tables shows other performance metrics such as Precision, Recall, and F1_Score.

**Table 7:** Proposed dataset.

Models	Accuracy (%)	Precision (%)	Recall (%)	F1 Score (%)
SVM	95.01	93.61	96.57	95.06
DT	92.90	93.33	92.35	92.84
KNN	95.14	92.33	98.42	95.27
GB	96.32	95.35	97.36	96.34
RF	97.11	96.85	97.36	97.11
**RaSK_GraDe**	98.03	97.89	98.15	98.02
**RaSK_GraDeL**	98.55	98.68	98.42	98.55

**Table 8:** PIMA dataset.

Models	Accuracy (%)	Precision (%)	Recall (%)	F1 Score (%)
SVM	79.00	79.21	79.21	79.21
DT	82.00	84.21	79.21	81.63
KNN	83.00	81.31	86.14	83.65
GB	84.50	85.00	84.16	84.58
RF	86.50	85.58	88.12	86.83
**RaSK_GraDe**	89.00	87.62	91.09	89.32
**RaSK_GraDeL**	86.50	88.54	84.16	86.29

**Table 9:** FHD dataset.

Models	Accuracy (%)	Precision (%)	Recall (%)	F1 Score (%)
SVM	95.64	96.63	94.85	95.73
DT	91.65	94.19	89.34	91.70
KNN	92.79	91.49	94.85	93.14
GB	95.83	95.96	95.96	95.96
RF	92.79	93.01	93.01	93.01
**RaSK_GraDe**	96.20	97.73	94.85	96.27
**RaSK_GraDeL**	96.20	97.37	95.22	96.28

**Table 10:** RTML with insulin dataset.

Models	Accuracy (%)	Precision (%)	Recall (%)	F1 Score (%)
SVM	94.29	89.47	100.00	94.44
DT	94.29	89.47	100.00	94.44
KNN	94.29	89.47	100.00	94.44
GB	97.14	94.44	100.00	97.14
RF	97.14	94.44	100.00	97.14
**RaSK_GraDe**	100.00	100.00	100.00	100.00
**RaSK_GraDeL**	100.00	100.00	100.00	100.00

### 4.3 Hyperparameter tuning

During the model setup of machine learning algorithms, certain strategies are employed to modify the parameter values in the most appropriate manner. The GridSearch method was used to identify the best appropriate parameter for the hyperparameter settings in these datasets. Table 11 provides the hyperparameter values for the machine learning methods used with the diabetes dataset.

**Table 11:** Hyperparameter tuning of models on (DHT) dataset.

Model	Parameter	Range	Best
SVM	Kernel	‘linear’, ‘Poly’, ‘rbf’	**rbf**
	C	0.01,0.1,1,10,100, 500,1000	**500**
	gamma	[1-10]	**1**
KNN	n_neighbors	[1-100]	**4**
	weights	‘uniform’, ‘distance’	**distance**
	algorithms	‘brute’, ‘ball_tree’, ‘kd_tree’, ‘auto’	**brute**
GB	learning_rate	0.001,0.01,0.1,0.2,0.3,0.4,0.5,	**0.1**
		0.6,0.7,0.8,0.9,1.0	
	max_depth	[1-6]	**3**
	n_estimators	10, 50, 100, 150, 200	**150**
RF	criterion	‘gini’, ‘entropy’	**entropy**
	class_weight	‘balance’, ‘auto’	**balance**
	max_depth	[1,10,16,20]	**16**
	max_features	[10,20,30,40,50]	**20**
	min_samples_leaf	[1-6]	**3**
	min_samples_split	[1-10]	**5**
	n_estimators	[10-50]	**32**
DT	criterion	‘gini’, ‘entropy’	**entropy**
	max_depth	[1,4,8,12,16,20]	**16**
	max_features	[10,20,30,40,50]	**20**
	min_samples_leaf	[1,3,6]	**3**
	min_samples_split	[0.0001,0.01,0.1,1,1.5]	**0.0001**
**RaSK_GraDe**	Voting	‘hard’, ‘soft’	**soft**
	Weights	[None, [2, 1, 1, 1, 1], [1, 1, 1, 3, 3], [1, 2, 2, 3, 3]]	**2, 1, 1, 1, 1]**
**RaSK_GraDeL**	Meta-Classifier	‘Logistic Regression’	**Logistic Regression**
	estimator	base-models	**base-models**

#### 4.3.1 Random search.

We employed random search as our strategy. Given that the hyperparameters are chosen at random, it is an excellent option for large data sets. Grid search, on the other hand, increases the computational cost by looking through every conceivable combination [58]. The mathematical expression of random search is given by equations 19 and 20. S is defined as an n-dimensional feasible region, x is a vector, and f is a real-valued function defined over S in equation (4). The objective is to find an x value in S that minimises f. These are the global optimal solutions, denoted by x’ and y’.

$$x^{\prime }=\mathrm{\backslash arg}\min_{x\in S}f(x)$$ (18)

$$y^{\prime }=f(x^{\prime })=\min_{x\in S}f(x)$$ (19)

Algorithm 5 presents the working of random search using cross validation techniques.

**Algorithm 5. Random search algorithm.**

**Require:** *y* is the new sample point; *x* is a random position.

1: Start

2: Let *x* be a random position in the search space.

3: **while** termination requirement not satisfied **do**

4:   Generate a new location *y* by sampling the hyper-sphere with a given radius around the current point *x*.

5:   **if** *f*(*x*)<*f*(*y*) **then**

6:    Update *x* = *y* to move to the new location.

7:   **end if**

8: **end while**

9: END =0

### 4.4 SHAPley Additive exPLANATIONS

A visualization tool called SHAPley Additive exPLANATIONS (SHAP) is used to improve the readability of the output produced by ML models. By calculating the relative contributions of each feature to the forecast, it can be used to explain the prediction of any model. A model’s output needs to be split into the sums of the impacts of each of its characteristics in order for SHAP to function. The contribution of each feature to the model result is represented by the value that SHAP yielded. These values can be used to help someone comprehend the significance of each component and to explain the model’s output. Businesses and teams who answer to clients or management would particularly benefit from this [59].

Fig 14, the feature names are arranged from top to bottom along the Y-axis. The amount of change in log odds is represented by the SHAP value, which is displayed on the X-axis. Each point on the graph is Coloured to indicate the value of the relevant attribute; red denotes high values and blue denotes low values. One row of data from the original dataset is represented by each point. Age, Glucose, Skin Thickness and Insulin are typically high and have a good SHAP value. this indicates that it has a beneficial impact on the result.

**Fig 12:**
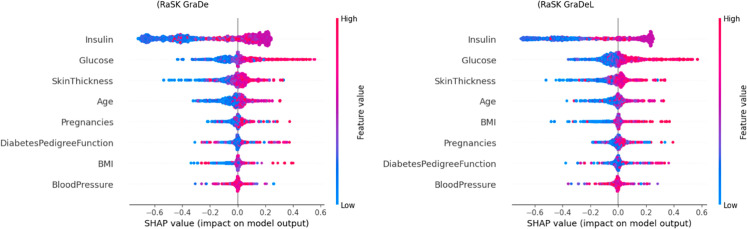
SHAP value impact on the Proposed (DHT) dataset using summary plot of ensemble model 1 (a). (RaSK_GraDe), and (b). (RaSK_GraDeL)

**Fig 13:**
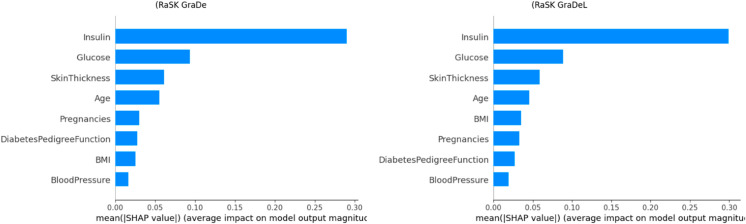
SHAP value impact on the Proposed (DHT) dataset using bar plot of ensemble model 1 (a). (RaSK_GraDe), and (b). (RaSK_GraDeL)

**Fig 14:**
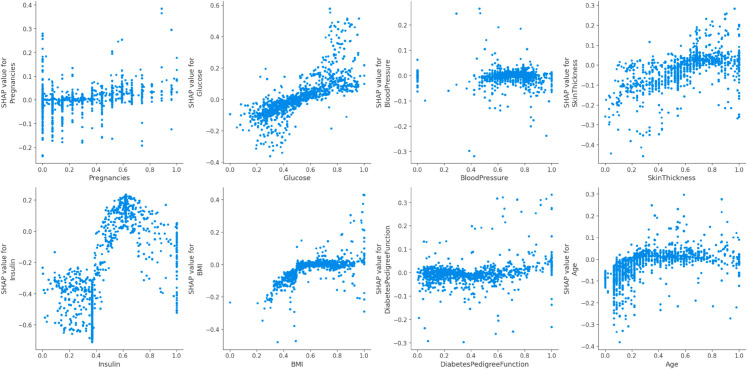
SHAP value impact on the Proposed (DHT) dataset using (RaSK_GraDe).

**Fig 15:**
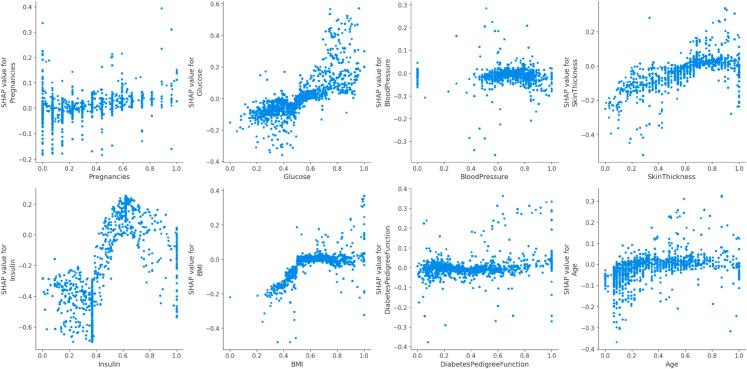
SHAP value impact on the Proposed (DHT) dataset using (RaSK_GraDeL).

Similarly, Fig 15, shows the same features high which means that both RaSK_Grade and RaSK_GraDeL have significant depend on these four features.

Figs 14 and 15 display the result of dependence of all features on the Outcome variable.We can see from the Fig 12 and 13, that the Glucose, and Insulin characteristics have high impact on the result as their combination increases the impact on the result also increases. This suggest that the higher value of these both features is beneficial for the model’s forecast.

## 5 Discussion

Although a great deal of study has been done on diabetes prediction, there is still opportunity for further development in this area. We use a merged dataset, as previously mentioned in the dataset information section, to predict diabetes in our work. We pre-processed the dataset once it was collected to prepare it for additional analysis. To predict diabetes, we used five supervised machine learning algorithms: RF, SVM, KNN, GB, and DT as a base models for the ensemble models (RaSK_GraDe and RaSK_GraDeL). Following the application of the ML techniques, we evaluated the outcomes using various performance metrics, including accuracy, precision, recall and F1-Score. The dataset we have used is never used in any other study before. Table VIII shows that our suggested ensemble models (RaSK_GraDe and RaSK_GraDeL) are quite good at predicting diabetes based solely on demographics characteristics. Furthermore, compared to the current models, the suggested ensemble models (RaSK_GraDe and RaSK_GraDeL) have additional validation metrics supporting it. The suggested ensemble models have some real-world applications in a variety of areas, including community health programs, telehealth and remote monitoring, personalised diabetes prevention plans, early diabetes risk assessment, and others. Furthermore, this research will aid in the development of personalized treatment and apps for managing diabetes. In summary, this study shows a wide range of possible practical uses in the medical field.

## 6 Conclusion and future directions

Diabetes remains a major global health concern. Beyond traditional clinical testing, data mining and machine learning are increasingly being used for early prediction. This study aimed to develop an automated model for diabetes prediction using five machine learning classifiers—Random Forest (RF), Support Vector Machine (SVM), K-Nearest Neighbors (KNN), Gradient Boosting (GB), and Decision Tree (DT)—alongside two ensemble models, RaSK_GraDe and RaSK_GraDeL.

The ensemble models outperformed individual classifiers, achieving accuracies of 98.03% and 98.55%, respectively, making them the most effective for diabetes prediction. The study also emphasized the importance of hyperparameter tuning via Random Search, as optimal hyperparameters significantly enhance model performance and generalization.

Future work should explore advanced ensemble techniques, deep learning approaches, dynamic hyperparameter tuning, and the integration of domain knowledge. Additionally, developing a user-friendly web and Android application for real-time diabetes prediction is recommended to support practical deployment.
